# RETRACTED: El-Bahr et al. Biosynthesized Iron Oxide Nanoparticles from *Petroselinum crispum* Leaf Extract Mitigate Lead-Acetate-Induced Anemia in Male Albino Rats: Hematological, Biochemical and Histopathological Features. *Toxics* 2021, *9*, 123

**DOI:** 10.3390/toxics14030198

**Published:** 2026-02-26

**Authors:** Sabry M. El-Bahr, Amal M. Elbakery, Nashwa El-Gazzar, Aziza A. Amin, Saad Al-Sultan, Mohammed A. Alfattah, Saad Shousha, Sameer Alhojaily, Mohammad Shathele, Islam I. Sabeq, Ahlam F. Hamouda

**Affiliations:** 1 Department of Biomedical Sciences, College of Veterinary Medicine, King Faisal University, Al-Ahsa 31982, Saudi Arabia; 2 Department of Biochemistry, Faculty of Veterinary Medicine, Alexandria University, Alexandria 21523, Egypt; 3 Department of Maize and Sugar Crop Disease Research, Plant Pathology Research Institute, ARC, Giza 12622, Egypt; 4 Department of Botany and Microbiology, Faculty of Science, Zagazig University, Zagazig 44519, Egypt; 5 Department of Histopathology, Faculty of Veterinary Medicine, Benha University, Benha 13736, Egypt; 6 Department of Public Health, College of Veterinary Medicine, King Faisal University, Al-Ahsa 31982, Saudi Arabia; 7 Camel Research Center, King Faisal University, Al-Ahsa 31982, Saudi Arabia; 8 Department of Physiology, Faculty of Veterinary Medicine, Benha University, Benha 13736, Egypt; 9 Department of Microbiology, College of Veterinary Medicine, King Faisal University, Al-Ahsa 31982, Saudi Arabia; 10 Department of Food Control and Hygiene, Faculty of Veterinary Medicine, Benha University, Benha 13736, Egypt; 11 Department of Forensic Medicine and Toxicology, Teaching Hospital, Faculty of Veterinary Medicine, Benha University, Benha 13736, Egypt

The journal retracts the article “Biosynthesized Iron Oxide Nanoparticles from *Petroselinum crispum* Leaf Extract Mitigate Lead-Acetate-Induced Anemia in Male Albino Rats: Hematological, Biochemical and Histopathological Features” [1] cited above.

Following publication, concerns were brought to the attention of the Editorial Office regarding the integrity of several figures presented in this publication [1].

Adhering to our standard procedure, an investigation was conducted by the Editorial Office and Editorial Board that identified partial duplications within Figures 4–6 within this article [1]. While the authors cooperated in the investigation, raw material meeting the journal’s original image requirements could not be provided for Editorial Board evaluation. Consequently, the Editorial Board has lost confidence in the reliability of the findings and has decided to retract this publication, as per MDPI’s retraction policy (https://www.mdpi.com/ethics#_bookmark30).

This retraction was approved by the Editor-in-Chief of the *Toxics* journal.

The authors did not agree to this retraction. 
